# Prevalence of insufficient physical activity among adult residents of Tehran: a cross-sectional report from Tehran Cohort Study (TeCS)

**DOI:** 10.1186/s12889-024-19201-6

**Published:** 2024-06-27

**Authors:** Shervin Mossavarali, Ali Vaezi, Amirhossein Heidari, Akbar Shafiee, Arash Jalali, Farshid Alaeddini, Soheil Saadat, Farzad Masoudkabir, Kaveh Hosseini, Ali Vasheghani-Farahani, Saeed Sadeghian, Mohamamdali Boroumand, Abbasali Karimi

**Affiliations:** 1 Tehran Heart Center, Cardiovascular Diseases Research Institute, Tehran University of Medical Sciences, Tehran, Iran; 2 Cardiac Primary Prevention Research Center, Cardiovascular Diseases Research Institute, Tehran University of Medical Sciences, Tehran, Iran; 3 Faculty of Medicine, Tehran Medical Sciences, Islamic Azad University, Tehran, Iran; 4 Department of Epidemiology and Biostatistics, School of Public Health, Tehran University of Medical Sciences, Tehran, Iran; 5 Department of Emergency Medicine, University of California, Irvine, CA USA; 6 Department of Cardiovascular Research, Tehran Heart Center, North Kargar Ave, Tehran, 1411713138 Iran

**Keywords:** Physical activity, Insufficient physical activity, Sedentary behavior, Tehran, Iran

## Abstract

**Background:**

Insufficient physical activity (PA) is a major risk factor for non-communicable diseases (NCDs) and one of the leading causes of premature mortality worldwide. This study examined the prevalence and independent determinants of insufficient PA among adults resident of Tehran utilizing Tehran Cohort Study Data (TeCS).

**Method:**

We used the recruitment phase data from the TeCS with complete data on PA. PA was assessed through a Likert-scaled question and categorized into three groups. Utilizing data from the 2016 national census, the age- and sex-weighted prevalence of insufficient PA in Tehran was determined. The adjusted logistic regression model is used to neutralize influencing factors and determine the factors associated with insufficient PA.

**Result:**

The weighted prevalence of insufficient PA was 16.9% among the 8213 adult citizens of Tehran, with a greater prevalence among females (19.0% vs. 14.8% among males). Additionally, older age groups, unemployed, housewives, and illiterate educated participants displayed a much higher prevalence of insufficient PA (*p* < 0.001). Moreover, Tehran’s central and southern districts had higher rates of insufficient PA. Concerning the adjusted regression model, older age (Odds ratio [OR]: 4.26, 95% confidence interval [95% CI]: 3.24–5.60, *p* < 0.001), a lower education level (*p* < 0.001), unemployment (OR: 1.80, 95% CI: 1.28–2.55, *p* = 0.001), being a housewife (OR: 1.44, 95% CI: 1.15–1.80, *p* = 0.002), higher body mass index (BMI) (OR for BMI > 30: 1.85, 95% CI: 1.56–2.18, *p* < 0.001), opium consumption (OR: 1.92, 95% CI: 1.46–2.52, *p* < 0.001), diabetes mellitus (OR: 1.25, 95% CI: 1.06–1.48, *p* = 0.008), hypertension (OR: 1.29, 95% CI: 1.11–1.50, *p* = 0.001), and coronary artery diseases (OR: 1.30, 95% CI: 1.05–1.61, *p* = 0.018), were significantly associated with insufficient PA.

**Conclusions:**

The identified associated factors serve as a valuable guide for policymakers in developing tailored intervention strategies to address the needs of high-risk populations, particularly among older adults and females.

**Supplementary Information:**

The online version contains supplementary material available at 10.1186/s12889-024-19201-6.

## Introduction

According to recent World Health Organization (WHO) guidelines on physical activity (PA) and sedentary behavior, insufficient PA, also known as physical inactivity or low PA, is fewer than 150 min of moderate-intensity aerobic PA or 75 min of vigorous-intensity aerobic PA per week or an equivalent combination of both for adults aged 18 to 64 [1]. Approximately one in three women and one in four men worldwide were insufficiently physically active in 2016, and the rate was higher for high-income countries [2]. As a critical worldwide issue, lack of adequate PA is strongly correlated with the risk of non-communicable diseases (NCDs), such as hypertension, type 2 diabetes mellitus (DM), breast cancer, colorectal cancer, chronic pain, and depression, estimated as the fourth leading cause of mortality which results in 5.3 million premature deaths each year [3]. Global healthcare costs directly associated with insufficient PA were estimated to be $54 billion in 2013, with another $14 billion correlated with lost productivity [4]. Additionally, 1–3% of national healthcare expenditures are attributed to inactivity [5].

Numerous studies have examined the prevalence of insufficient PA and its evolution over time in developing regions, such as the Middle East and North Africa [6, 7]. Moreover, several investigations have estimated that there is insufficient PA in Iran as a developing country. Regarding recent reports, the prevalence of insufficient PA in Iran is about 51.3% for the entire population [8]. In addition, the prevalence of insufficient PA in Iranian adults over 18 has doubled from 2001 to 2016, which means more than half of the population had insufficient PA as of 2016 [9]. Further, estimates for insufficient PA were more prevalent among females and ranged from 39.5 to 78.7% among Iranian cities, showing a noticeable difference among Iranian regions. Moreover, insufficient PA accounted for about 3.7% of all-cause mortalities in Iran in 2019 [10].

Even though Tehran is Iran’s capital and most densely populated city, and there are changes in the number of growing dwellers, few epidemiological studies have addressed the prevalence of insufficient PA. Moreover, it should be considered that insufficient PA poses a serious health risk for general populations, particularly among older adults, and existing reports are nearly outdated, and their samples did not cover all districts [11, 12]. In light of this issue, a comprehensive report on the insufficient PA status among Tehran residents is required to establish proper health-related management and policies. Therefore, we sought to estimate PA levels among adult residents of Tehran with a focus on sex and age subgroups regarding the Tehran Cohort Study (TeCS) data.

## Methods

### Study design and participants

We retrieved data from the recruitment phase of the TeCS in this cross-sectional study. The details of the TeCS protocol have been explained previously [13]. Briefly, TeCS is a longitudinal cohort study in Tehran aiming to identify the incidence, prevalence, and trend of cardiovascular diseases and their associated risk factors in a random sample of Tehran’s adult population. Between March 2016 and March 2019, 4,215 households consisting of 8,296 adults 35 years or older were enrolled in the TeCS data bank. Several demographic and medical data were obtained, including past medical history, medications, and familial history, as well as anthropometric and physiological measurements, biochemistry, and laboratory tests. During this analysis, we selected participants with complete PA data. The Research Council and Medical Ethics Committee at Tehran University of Medical Sciences ratified the protocols of TeCS (IR.TUMS.MEDICINE.REC.1399.074). All participants signed the informed consent form upon enrollment. Moreover, in the case of members, they may not be capable of providing informed consent for their participation (Illiterate), or in such instances, the legal guardian or an appropriate representative of these participants provided informed consent on their behalf.

### Data collection and measurements

Predesigned checklists were developed to assess demographic information, occupation, marital status, ethnicity, education level, preexisting comorbidities, and metabolic risk factors. Furthermore, an experienced nurse measured anthropometric indices, including weight, height, body mass index (BMI), waist circumference, and hip circumference. BMI was expressed as weight fractioned by height square (kg/m2) [14]. The hip was metered around the widest part of the buttocks and waist circumferences at the midpoint between the lower margin of the lowest palpable rib and the top of the iliac crest [15]. Fasting venous blood samples were collected after 8–12 h of fasting to measure fasting blood glucose (FBG), creatinine, and lipid profiles. Furthermore, a digital brachial cuff sphygmomanometer was used to measure blood pressure after at least five minutes of rest in a sitting position. In addition, as part of our in-person interview checklist, prior to answering, our expert nurse completely explained each part of the study form based on the subject level of education about the question and the exact meaning of each score. In the case of physical activity, we utilized a subjective five-score Likert scale. The term “inactive” was applied to individuals who, for any reason, lacked the ability to move. “Low activity” described those who were capable of movement but did not engage in any walking activities beyond basic necessities, such as going to the restroom or kitchen. “Moderate activity” refers to individuals who engage in walking as part of their daily routines, such as shopping or going to the park. An “active” individual was someone who, in addition to walking, participated in simple and amateur sports sessions up to two or three times a week. Finally, the “very active” category was designated for those who engaged in professional and regular sports activities on a daily basis. Finally, all subjects were classified as average daily PA in three levels, including low, intermediate, and high activity.

### Definitions

Defining the terms tobacco use, opium and alcohol consumption, and glycemic conditions, as well as preexisting comorbidities such as chronic kidney diseases (CKD) and coronary artery disease (CAD), were comprehensively expounded in our previous studies [16, 17]. Briefly, current tobacco use is defined as the daily or occasional smoking of cigarettes, pipes, or hookahs [17]. Quitted tobacco users were those who had quit smoking one month before the interview. The definition of opium consumption was any use of opium or its derivatives during the previous year. Alcohol use was labeled as drinking any alcoholic beverages in the previous year. Moreover, DM is defined by previously diagnosed type 2 diabetes by healthcare providers, or FBG over 126 mg/dl following an overnight fast of 8–12 h or taking glucose-lowering medications such as oral hypoglycemic agents or insulin. The condition of impaired fasting glucose was identified as an FBS level spanning from 100 to 125 mg/dl in individuals without a prior diagnosis of diabetes or utilizing any glucose-lowering drugs [18]. Hypertension is also described as a self-reported previous diagnosis of hypertension or measurements showing systolic blood pressure (BP) of 140 mmHg or higher, as well as diastolic BP of 90 mmHg or higher, or the use of antihypertensive drugs [19]. Systolic BP between 130 and 140 mmHg and/or Diastolic BP between 85 and 90 mmHg were classified as high normal blood pressure. Dyslipidemia is classified to suboptimal levels of high-density lipoprotein (HDL) cholesterol and/or increased levels of triglyceride or low-density lipoprotein (LDL) cholesterol, along with the usage of lipid-lowering medications.

### Statistical analysis

Normally distributed continuous variables were described as mean with standard deviation (SD) and were compared between the PA levels using a One-way analysis of variance (ANOVA) test. Skewed distributed continuous variables were expressed as median with 25th and 75th percentiles (interquartile range boundaries) and were compared between PA levels applying the Kruskal-Wallis test. The normality of the continuous variables was assessed using descriptive measures as well as histogram charts. Categorical variables were presented as the frequency with percentages and compared between PA levels using the Chi-square test. The adjusted association between low PA and baseline covariates was assessed using a logistic regression model. Age, gender, marital status, level of education, ethnicity, occupation, tobacco, opium, and alcohol use, BMI, Glycemic condition, hyperlipidemia, hypertension, chronic kidney disease, coronary artery diseases, and family history of coronary artery diseases were all adjusted for. Their adjusted association of the variables with low effects was reported via odds ratio (OR) with a 95% confidence interval (CI). The calibration of the logistic regression model was assessed utilizing the Hosmer-Lemeshow goodness of fit test. Also, the multicollinearity of the multivariable model was evaluated using the variance inflation factors (VIFs) for each covariate using the command *collin* in Stata statistical software, release 14.2 (College Station, TX: Stata Corp LP.). The sex and age-weighted standardized prevalence of PA levels, based on their age distribution, among the grownup citizens of Tehran was calculated according to the Iran Population and Housing Census 2016 [20] and provided a 95% confidence interval (CI). The frequency of low levels of PA in different regions of Tehran was drawn based on the initial three digits of the postal code using the *spmap* module in Stata statistical software, release 14.2 (College Station, TX: Stata Corp LP.). For other analyses, we used STATA version 14.2 (College Station, TX: Stata Corp LP). Statistical significance was attributed to p-values lower than 0.05.

## Results

### General characteristics of the study population

**Fig. 1:**
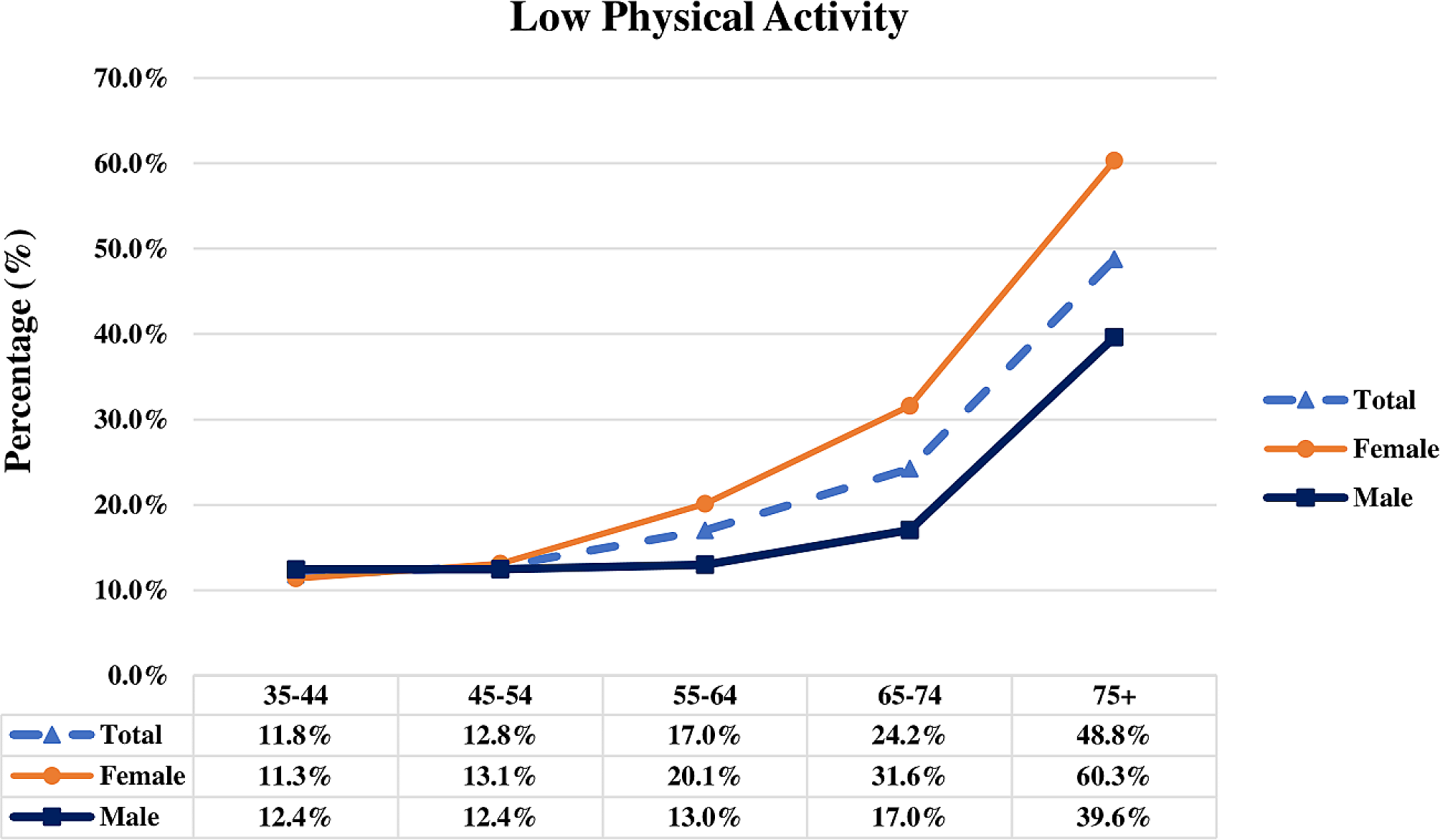
The percentage of individuals with low physical activity based on age and sex

Out of 8,296 participants, 8213 adults met our study inclusion criteria (females: 54.1%; mean age: 53.7 ± 12.7 years), and 83 (1.0%) had missing or incomplete data on their PA and were excluded from the final analysis. As illustrated in Tables 1 and 1449 (17.4%) of participants reported low PA levels (females: 59.6%) and tended to be older in comparison with other groups (*p* < 0.001). In the details, low-active had the lowest and highest prevalence among the age group of 35–44 years (11.8%) and > 75 (48.8%), respectively. The most significant jump in low PA reports was seen between the age group of 64–75 years and > 75 years (Fig. 1). Also, the age pattern of low PA prevalence was affected by gender. A higher proportion of low-active participants were female, while a higher proportion of high-active participants were male (*p* < 0.001). The low-active group has a higher percentage of individuals with lower education levels (illiterate and 1–5 years of education) and a lower percentage with higher education levels (> 12 years) than the other groups (*p* < 0.001). In addition, most participants of the low-active group were not employed (72%, *p* < 0.001) than their peers. Moreover, there was a statistically higher prevalence of opium consumption, diabetes, hypertension, dyslipidemia, CKD, and CAD in the low-active group compared with other groups (*p* < 0.001). Comparative to those with a moderate or high level of activity, low-active individuals had a considerable elevation in BMI, with the highest percentage of individuals with a BMI > 30, a larger waist and hip circumference, as well as higher levels of systolic and diastolic BP values (*p* < 0.001). According to laboratory measurements, the low-active group also had higher FBG, urea, creatinine, and triglycerides levels than their peers in other groups (*p* < 0.001). The percentage of participants’ physical activity levels regarding age and sex are detailed in Table S1.

**Table 1:** Baseline characteristics of the study population according to physical activity levels

**Characteristics** ^**#**^	**All participants (** ***n*** ** = 8213)**	**Low active participants (** ***n*** ** = 1449)**	**Intermediate active participants (** ***n*** ** = 4764)**	**High active participants (** ***n*** ** = 2000)**	P-value*	
**Age, year**	53.7 ± 12.73	59.7 ± 14.7	53 ± 12.13	51.2 ± 11.16	< 0.001	
**Age group, year**					< 0.001	
35–44	2311 (28.1)	273 (18.8)	1400 (29.4)	638 (31.9)		
45–54	2193 (26.7)	281 (19.4)	1296 (27.2)	616 (30.8)		
55–64	1944 (23.7)	330 (22.8)	1159 (24.3)	455 (22.8)		
65–74	1205 (14.7)	292 (20.2)	673 (14.1)	240 (12)		
>75	560 (6.8)	273 (18.8)	236 (5)	51 (2.6)		
**Gender**					< 0.001	
Female	4447 (54.1)	863 (59.6)	2688 (56.4)	896 (44.8)		
Male	3766 (45.9)	586 (40.4)	2076 (43.6)	1104 (55.2)		
**Marital Status**					0.272	
Single	67 (0.8)	12 (0.8)	39 (0.8)	16 (0.8)		
Married	7945 (96.8)	1413 (97.5)	4597 (96.5)	1935 (96.9)		
Other (Divorced/Widowed/…)	198 (2.4)	24 (1.7)	128 (2.7)	46 (2.3)		
**Education, year**					< 0.001	
Illiterate	581 (7.1)	251 (17.3)	296 (6.2)	34 (1.7)		
1–5	835 (10.2)	194 (13.4)	508 (10.7)	133 (6.7)		
6–12	4275 (52.1)	656 (45.3)	2578 (54.1)	1041 (52.2)		
>12	2516 (30.7)	347 (24)	1382 (29)	787 (39.4)		
**Ethnicity**					0.004	
Fars	3995 (48.7)	736 (50.9)	2348 (49.3)	911 (45.7)		
Azari	2438 (29.7)	435 (30.1)	1397 (29.3)	606 (30.4)		
Other	1773 (21.6)	276 (19.1)	1019 (21.4)	478 (24)		
**Occupation**					< 0.001	
Employed	3363 (41)	407 (28.1)	1872 (39.3)	1084 (54.3)		
Housewife	3187 (38.8)	692 (47.8)	1967 (41.3)	528 (26.5)		
Retired	1427 (17.4)	281 (19.4)	805 (16.9)	341 (17.1)		
Unemployed	231 (2.8)	69 (4.8)	120 (2.5)	42 (2.1)		
**Tobacco use**					< 0.001	
Never	6306 (76.8)	1135 (78.3)	3712 (78)	1459 (73)		
Quitted	333 (4.1)	77 (5.3)	176 (3.7)	80 (4)		
Yes	1571 (19.1)	237 (16.4)	874 (18.4)	460 (23)		
**Alcohol consumption**					< 0.001	
No	7467 (91.3)	1332 (92.1)	4421 (93)	1714 (86.3)		
Yes	716 (8.7)	114 (7.9)	331 (7)	271 (13.7)		
**Opium consumption**					< 0.001	
No	7755 (94.7)	1336 (92.5)	4526 (95.2)	1893 (95.1)		
Yes	433 (5.3)	108 (7.5)	228 (4.8)	97 (4.9)		
**Glycemic condition**					< 0.001	
Normal	4523 (56)	678 (47.6)	2636 (56.3)	1209 (61.4)		
Impaired fasting glycemia (Prediabetes)	2059 (25.5)	355 (24.9)	1174 (25.1)	530 (26.9)		
Diabetes mellitus	1489 (18.4)	391 (27.5)	869 (18.6)	229 (11.6)		
**Hypertension**						
No	5906 (71.9)	817 (56.4)	3469 (72.8)	1620 (81)	< 0.001	
Yes	2305 (28.1)	631 (43.6)	1294 (27.2)	380 (19)		
**Dyslipidemia**					< 0.001	
No	5532 (67.4)	857 (59.2)	3199 (67.1)	1476 (73.8)		
Yes	2679 (32.6)	590 (40.8)	1565 (32.9)	524 (26.2)		
**Chronic kidney disease**						
No	8142 (99.1)	1419 (97.9)	4733 (99.3)	1990 (99.5)	< 0.001	
Yes	71 (0.9)	30 (2.1)	31 (0.7)	10 (0.5)		
**Coronary artery diseases**						
No	7578 (92.3)	1251 (86.3)	4424 (92.9)	1903 (95.2)	< 0.001	
Yes	634 (7.7)	198 (13.7)	340 (7.1)	96 (4.8)		
**Family history of coronary artery diseases, n (%)**					0.318	
No	7441 (90.6)	1328 (91.6)	4307 (90.4)	1806 (90.3)		
Yes	772 (9.4)	121 (8.4)	457 (9.6)	194 (9.7)		
**Systolic BP, mmHg**	121.8 ± 18.85	125.5 ± 20.35	121.4 ± 18.77	119.9 ± 17.51	< 0.001	
**Diastolic BP, mmHg**	80.8 ± 10.83	81 ± 11.79	81 ± 10.76	80.1 ± 10.21	< 0.001	
**BMI, kg/m** ^**2**^	28 ± 4.83	29.3 ± 5.67	28 ± 4.67	27 ± 4.28	< 0.001	
**BMI category, kg/m** ^**2**^					< 0.001	
<25	2283 (28)	325 (23.1)	1285 (27.1)	673 (33.7)		
25-29.9	3396 (41.7)	486 (34.5)	2017 (42.5)	893 (44.8)		
>30	2466 (30.3)	596 (42.4)	1441 (30.4)	429 (21.5)		
**Waist circumference, cm**	96.3 ± 11.77	100.8 ± 12.95	96.1 ± 11.4	93.6 ± 10.78	< 0.001	
**Hip circumference, cm**	105.2 ± 9.9	108.4 ± 11.82	105.1 ± 9.53	103.2 ± 8.66	< 0.001	
**Waist / Hip ratio**	0.92 ± 0.072	0.93 ± 0.076	0.91 ± 0.072	0.91 ± 0.07	< 0.001	
**Fasting blood glucose, mg/dl**	97 [90, 107]	99 [91, 113]	97 [90, 108]	96 [90, 104]	< 0.001	
**Urea, mg/dl**	27.7 [22.8, 33.7]	29.1 [23.8, 36.8]	27.1 [22.4, 33]	28 [23, 33.7]	< 0.001	
**Creatinine, mg/dl**	0.8 [0.7, 0.94]	0.82 [0.7, 0.99]	0.8 [0.69, 0.92]	0.82 [0.71, 0.95]	< 0.001	
**Triglyceride, mg/dl**	124 [88, 175]	129 [93.5, 182]	126 [88, 178]	117 [83, 167]	< 0.001	
**Total cholesterol, mg/dl**	170 [145, 197]	171 [141, 198.5]	169 [144, 196]	172 [149, 198]	0.017	
**HDL, mg/dl**	43 [36, 52]	43 [35, 51]	43 [36, 51]	44 [37, 53]	0.001	
**LDL, mg/dl**	111 [90, 134]	109 [85, 134]	110 [90, 134]	113 [92, 137]	< 0.001	

BMI: Body Mass Index; BP: Blood Pressure; HDL: High-Density Lipoprotein; LDL: Low-Density Lipoprotein

^#^ Data are presented as mean ± standard deviation and median [interquartile range] for continuous and number (Percentage calculated for rows) for categorical variables.

* *P* < 0.05 was considered significant.

### Age and gender distribution of insufficient physical activity

**Fig. 2:**
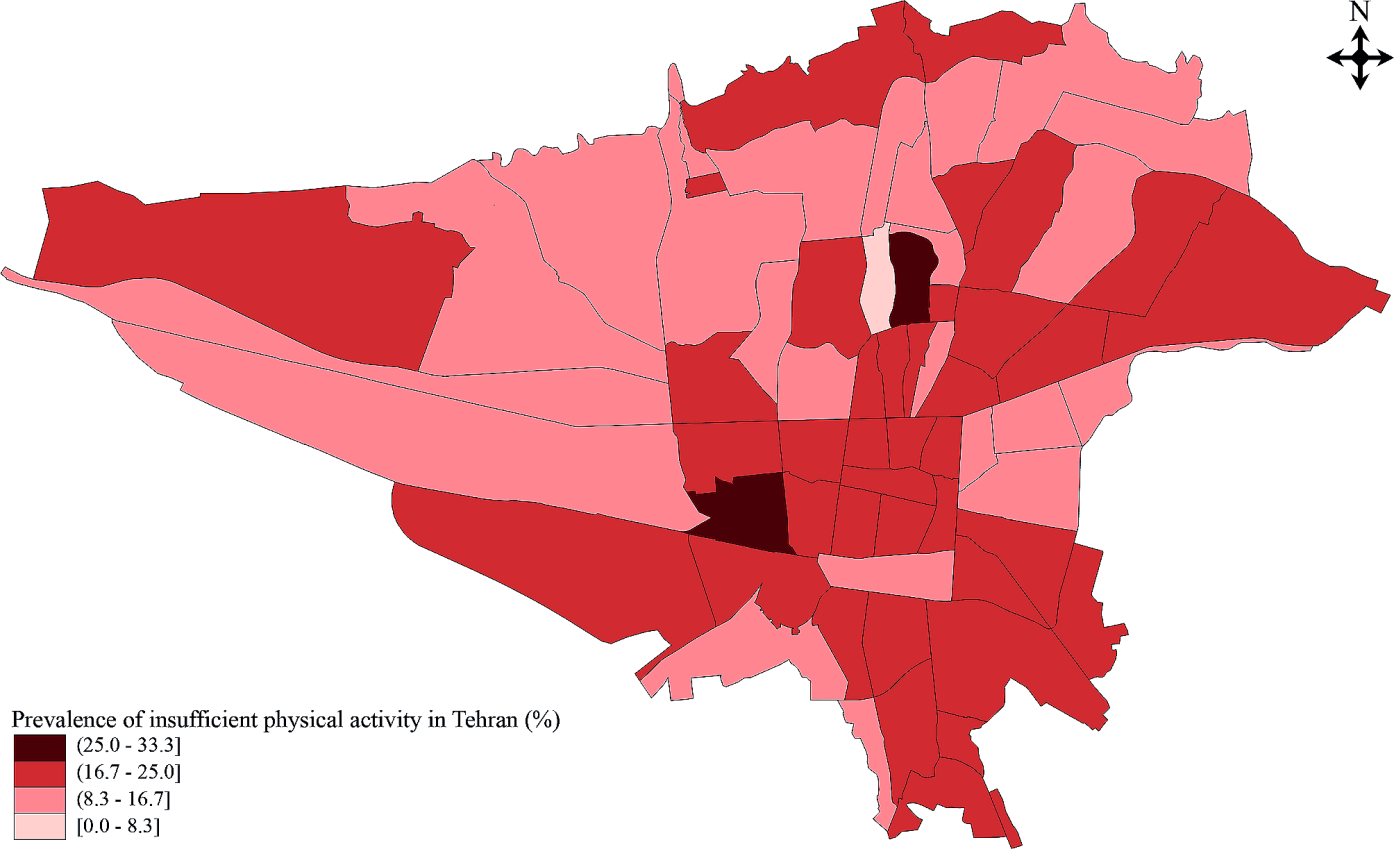
The geographical distribution of insufficient physical activity among Tehran districts

The age and gender-weighted prevalence of low PA was 16.9% (95% CI: 15.2–18.6) among citizens of Tehran aged 35 years or older. The age-weighted prevalence of insufficient PA was 14.8% (95% CI: 12.5–17.4) in males and 19.0% (95% CI: 16.6–21.4) in females. As depicted in Fig. 2, people in Tehran’s central and eastern districts were more physically inactive.

### Determinants associated with insufficient physical activity

Regarding insufficient PA determinants, individuals in the 65–74 age group have 1.46 times higher odds of being low active than those in the 35–44 age group (95% CI: 1.15–1.84, *p* = 0.002). Further, participants aged more than 75 have the highest odds of being insufficiently active (OR:4.26, 95% CI: 3.24–5.60, *p* < 0.001). Interestingly, there was an inverse association between education and low PA. By detail, individuals with 1–5, 6–12, and over 12 years of education have 46% (95% CI: 0.42–0.70), 55% (95% CI: 0.36–0.56), and 47% (95% CI: 0.41–0.69) lower odds of being in the low active group, respectively (*p* < 0.001). In addition, lower PA had a statistically significant correlation with unemployment and being a housewife (OR: 1.80, 95% CI: 1.28–2.55 *p* = 0.001; OR: 1.44, 95% CI: 1.15–1.80 *p* = 0.002, respectively), opium consumption (OR: 1.92, 95% CI: 1.46–2.52, *p* < 0.001), hypertension (OR: 1.29, 95% CI: 1.11–1.50, *p* = 0.001), and CAD (OR: 1.30, 95% CI: 1.05–1.61, *p* = 0.018). Moreover, the odds of insufficient PA in obese participants (BMI > 30) were 85% higher than in healthy people (BMI < 25) (OR for BMI > 30: 1.85, 95% CI: 1.56–2.18, *p* < 0.001). In the case of the glycemic condition, a strong relationship was found between the presence of diabetes mellitus and lower PA (OR:1.25, 95% CI: 1.06–1.48, *p* = 0.008). Further, no considerable connection was identified between lower PA and gender, marital status, ethnicity, and alcohol consumption. Table 2 comprehensively depicts the correlation between the study variables and the low level of PA.

**Table 2:** Multivariable analysis of risk factors associated with insufficient physical activity among our study participants

Characteristics	Adjusted OR (95% CI)	*P*-value*
**Age (year)**		
35–44	Ref.	< 0.001
45–54	0.93 (0.77–1.12)	0.456
55–64	1.09 (0.89–1.34)	0.398
65–74	1.46 (1.15–1.84)	0.002
>75	4.26 (3.24–5.60)	< 0.001
**Male sex**	0.94 (0.76–1.16)	0.56
**Marital Status**		
Single	Ref.	0.589
Married	0.85 (0.44–1.65)	0.626
Other (Divorced/Widowed/…)	0.68 (0.31–1.53)	0.357
**Education**		
Illiterate	Ref.	< 0.001
1–5	0.54 (0.42–0.70)	< 0.001
6–12	0.45 (0.36–0.56)	< 0.001
>12	0.53 (0.41–0.69)	< 0.001
**Ethnicity**		
Fars	Ref.	0.429
Azari	0.92 (0.79–1.06)	0.247
Other	0.93 (0.79–1.09)	0.346
**Occupation**		
Employed	Ref.	< 0.001
Housewife	1.44 (1.15–1.80)	0.002
Retired	0.98 (0.80–1.20)	0.825
Unemployed	1.80 (1.28–2.55)	0.001
**Tobacco Use**		
Never	Ref.	0.979
Quitted	1.00 (0.73–1.37)	0.983
Yes	0.98 (0.81–1.19)	0.844
**Alcohol consumption**	1.14 (0.89–1.45)	0.302
**Opium consumption**	1.92 (1.46–2.52)	< 0.001
**BMI (kg/m** ^**2**^ **)**		
<25	Ref.	< 0.001
25-29.9	1.05 (0.89–1.24)	0.564
>30	1.85 (1.56–2.18)	< 0.001
**Glycemic condition, n (%)**		
Normal	Ref.	0.03
Impaired fasting glycemia (Prediabetes)	1.05 (0.90–1.22)	0.517
Diabetes mellitus	1.25 (1.06–1.48)	0.008
**Dyslipidemia**	1.01 (0.87–1.16)	0.919
**Hypertension**	1.29 (1.11–1.50)	0.001
**Chronic kidney disease**	1.35 (0.78–2.36)	0.289
**Coronary artery diseases**	1.30 (1.05–1.61)	0.018
**Family history of coronary artery diseases**	0.92 (0.74–1.15)	0.472

BMI: Body Mass Index; CI: Confidence interval; OR: Odds ratio

Hosmer-Lemeshow goodness of fit test: P-value: 0.275

All variance inflation test (VIF) values were less than 2, indicating no significant multicollinearity concerns.

* *P* < 0.05 was considered significant.

## Discussion

In the current study, we observed that the weighted prevalence of insufficient PA among Tehran adult citizens is about 16.9%, with a higher prevalence in female participants (19.0% vs. 14.8% in men). Moreover, we determined older age, lower education level, unemployment, being a housewife, higher BMI, and opium use, as well as the presence of DM, hypertension, and CAD, were independent determinants of insufficient PA among our study population.

Few studies have investigated insufficient PA in the different geographical areas of Iran. As reported by the Tehran Lipid and Glucose Study (TLGS), approximately half of the Tehran population experienced insufficient PA from Phase II to Phase IV during seven years of follow-up, with rates at 45.9% and 42.6%, respectively [11]. Nonetheless, their study was carried out within a single district of Tehran with identical socioeconomic backgrounds and smaller sample sizes compared to our study. Another investigation performed in Kurdistan province utilized data collected from individuals aged 15 to 64 years [21]. Based on their results, insufficient PA increased from 16.9 to 26.8% within a 4-year follow-up period. A similar investigation conducted in Kerman Province indicated that 47.2% of 3416 adult participants reported low PA levels [22]. However, recent results from the STEP survey study concerning PA among all regions of Iran expounded that Yazd province exhibited the highest prevalence of insufficient PA (63.45%), whereas West Azerbaijan province displayed the lowest prevalence (39.53%) [8]. In accordance with STEP survey results, other recent national studies have indicated that the incidence of sedentary behavior among Iranian adults surpasses the global average and reported a higher prevalence of insufficient PA compared to the present study. [23–25]. These differences in results among studies may be explained by variations in the methodology utilized for gathering data on PA as well as discrepancies in the operational definition of the term “insufficient PA.” Furthermore, it appears that the citizens of Tehran exhibited a higher level of activity in the execution of their daily routine.

Interestingly, the present study revealed that Tehran’s lower socioeconomic districts exhibited a higher prevalence of insufficient PA. It seems that the level of socioeconomic status is inversely related to PA. This result is in accordance with other reports from developed countries, especially when education is considered [26–28]. However, a recent study in western Iranian cities found no significant correlation between PA and socioeconomic status [29]. Nonetheless, it can be observed that with advancement in socioeconomic status, an increasing amount of time is devoted to engaging in physically active leisure activities.

Moreover, participants aged 35 to 44 demonstrated the least amount of insufficient PA, whereas those 75 years of age and older depicted the greatest levels. Consistent with previous studies, there is an inverse relationship between age and low PA, which has been identified as a significant determinant of PA [30–33]. Apparently, with increasing age, older people seem to be less motivated to be physically active and prefer quiet activities. Additionally, the presence of different comorbidities among the older adult can negatively affect their daily activities.

Unlike prior studies suggesting a correlation between gender and insufficient PA, our study found no remarkable relationship [23, 30, 33, 34]. Concerning this finding, gender-based cultural and social barriers appear to have been mostly dismantled in Tehran, and women are involved in more physically active occupations and positions. With this trend, it is expected that this gap will gradually narrower and narrower over time.

The current study corroborates a positive two-way relationship between sedentary behavior and BMI. Additional investigations have also discovered an inverse association linking BMI and PA [35, 36]. Nevertheless, some studies have failed to support this correlation [37, 38]. Overall, it seems that engaging in PA is followed by an increase in muscle mass rather than total body fat, which leads to a decrease in BMI [39].

Based on prior studies, the association between insufficient PA and tobacco and alcohol consumption has also been controversial. While several studies have suggested that smoking can lead to less PA, other studies have expounded a correlation between occasional smoking and increased levels of PA [12, 25, 40]. However, in our study, we observed no significant association between insufficient PA and tobacco or alcohol use.

Clinical and epidemiological evidence indicates that higher levels of PA are associated with reduced risk for CAD, DM hypertension, and dyslipidemia [41–43]. As an observational study, the current study cannot prove a causal relationship; however, our results align with other studies, illustrating that increased PA is associated with lower occurrences of DM, hypertension, and CAD.

Given the increasing prevalence of non-communicable diseases, it is imperative to formulate strategies that promote greater PA among individuals. Government authorities and policy creators should proactively offer increased incentives and opportunities for adults, particularly the elderly population, to partake in everyday activities and actively seek methods to reduce their sedentary periods. This is essential for the preservation of their holistic well-being and health. Furthermore, our study provides valuable insights into the associations between variables and insufficient PA within our study participants, so future investigations should be conducted with larger sample sizes and longitudinal follow-up to evaluate specific and causal associations more comprehensively. Such studies would enhance the external validity and applicability of the findings, allowing for broader generalization across different populations and settings. This future research is essential to confirm and extend our results, thereby providing a more robust understanding of the factors influencing insufficient PA.

### Strengths and limitations

This study is particularly noteworthy for being the initial survey to examine the prevalence of insufficient PA in a broad, diverse sample across all regions of Tehran. Nevertheless, it has its limitations. First, the study’s cross-sectional nature cannot establish the causal relationship between PA and its correlates. Next, while surveys are frequently utilized in cross-sectional and epidemiological research, there is a possibility for either underestimation or overestimation of the gathered information. Third, the participants’ PA level was assessed using a Likert-scaled self-report question, which cannot be relied upon as an entirely accurate measure. To overcome this shortcoming, we are using a detailed questionnaire on daily PA in the first follow-up of TeCS that would complete the findings of the current analysis.

## Conclusion

The prevalence of insufficient PA was found to be about half of the reported global statistics among adult citizens of Tehran. However, the older age groups showed a much higher prevalence of insufficient PA, and it is necessary to prompt decisions for improving this situation in the future. We also determined unemployment, being a housewife, illiterate education, higher BMI, DM, hypertension, CAD, and opium use as independent determinants of PA. Policymakers can use these results as a basis to develop tailored intervention strategies aimed at providing for the requirements of high-risk individuals. It is also imperative that the nation establishes a policy to encourage PA to improve its citizens’ well-being and reduce sedentariness. Moreover, we recommend future studies with larger sample sizes and longitudinal follow-up to evaluate specific and causal associations more thoroughly. These investigations are crucial to confirm and expand upon our results, providing a more comprehensive understanding of the factors influencing insufficient PA.

### Electronic supplementary material

Below is the link to the electronic supplementary material.

Supplementary Material 1: Table S1: Percentage of participants’ physical activity levels based on age and sex

## Data Availability

All the data generated or analyzed during the current study are available from the corresponding author upon reasonable request.
