# Supplementary material for: Prevalence of insufficient physical activity among adult residents of Tehran: a cross-sectional report from Tehran Cohort Study (TeCS)
Source: BMC Public Health. 2024 Jun 27;24:1722. doi: 10.1186/s12889-024-19201-6 (PMC11212377; doi:10.1186/s12889-024-19201-6)
Supplement: Supplementary file 1 — Supplementary Material 1: Table S1: Percentage of participants’ physical activity levels based on age and sex [file 12889_2024_19201_MOESM1_ESM.docx]

| **Table S1.** Percentage of participants’ physical activity levels based on age and sex. | | | |
| --- | --- | --- | --- |
| **Age groups** | **Low active group (%)** | **Intermediate active group (%)** | **High active group (%)** |
| **Total** |  |  |  |
| 35-44 | 11.80% | 60.60% | 27.60% |
| 45-54 | 12.80% | 59.10% | 28.10% |
| 55-64 | 17.00% | 59.60% | 23.40% |
| 65-74 | 24.20% | 55.90% | 19.90% |
| >75 | 48.80% | 42.10% | 9.10% |
| **Female** |  |  |  |
| 35-44 | 11.30% | 65.40% | 23.20% |
| 45-54 | 13.10% | 61.70% | 25.20% |
| 55-64 | 20.10% | 61.80% | 18.10% |
| 65-74 | 31.60% | 54.80% | 13.60% |
| >75 | 60.30% | 35.60% | 4.00% |
| **Male** |  |  |  |
| 35-44 | 12.40% | 54.20% | 33.40% |
| 45-54 | 12.40% | 56.00% | 31.50% |
| 55-64 | 13.00% | 56.80% | 30.30% |
| 65-74 | 17.00% | 56.90% | 26.10% |
| >75 | 39.60% | 47.30% | 13.10% |
